# Networks of fibers and factors: regulation of capsule formation in *Cryptococcus neoformans*

**DOI:** 10.12688/f1000research.8854.1

**Published:** 2016-07-22

**Authors:** Hao Ding, François L. Mayer, Eddy Sánchez-León, Glauber R. de S. Araújo, Susana Frases, James W. Kronstad

**Affiliations:** 1 Michael Smith Laboratories, University of British Columbia, Vancouver, Canada; 2 Laboratório de Ultraestrutura Celular Hertha Meyer, Instituto de Biofísica Carlos Chagas Filho, Federal University of Rio de Janeiro, Rio de Janeiro, Brazil; 3 Department of Microbiology and Immunology, University of British Columbia, Vancouver, Canada

**Keywords:** Fungi, cryptococcal capsule, transcription factors

## Abstract

The ability of the pathogenic fungus *Cryptococcus neoformans* to cause life-threatening meningoencephalitis in immunocompromised individuals is due in large part to elaboration of a capsule consisting of polysaccharide fibers. The size of the cell-associated capsule is remarkably responsive to a variety of environmental and host conditions, but the mechanistic details of the regulation, synthesis, trafficking, and attachment of the polysaccharides are poorly understood. Recent studies reveal a complex network of transcription factors that influence capsule elaboration in response to several different signals of relevance to disease (e.g., iron deprivation). The emerging complexity of the network is consistent with the diversity of conditions that influence the capsule and illustrates the responsiveness of the fungus to both the environment and mammalian hosts.

## Introduction

Fungal pathogens pose an under-appreciated threat to human health, and the incidence of invasive fungal infections is on the rise. This is particularly alarming given that diseases caused by fungi are difficult to treat owing to a limited selection of antifungal drugs and emerging resistance ^1, 2^. Cryptococcal meningoencephalitis caused by the yeast *Cryptococcus neoformans* is one of the most prevalent invasive fungal diseases ^3^. In fact, it is estimated that there are ~1 million cases of cryptococcal meningoencephalitis per year globally, resulting in >600,000 deaths, with the greatest occurrence in the immunocompromised HIV/AIDS population in sub-Saharan Africa ^4^. In addition, the related species *Cryptococcus gattii* has emerged as a pathogen of people who are considered to be immunocompetent ^5– 7^.

**Figure 1.**
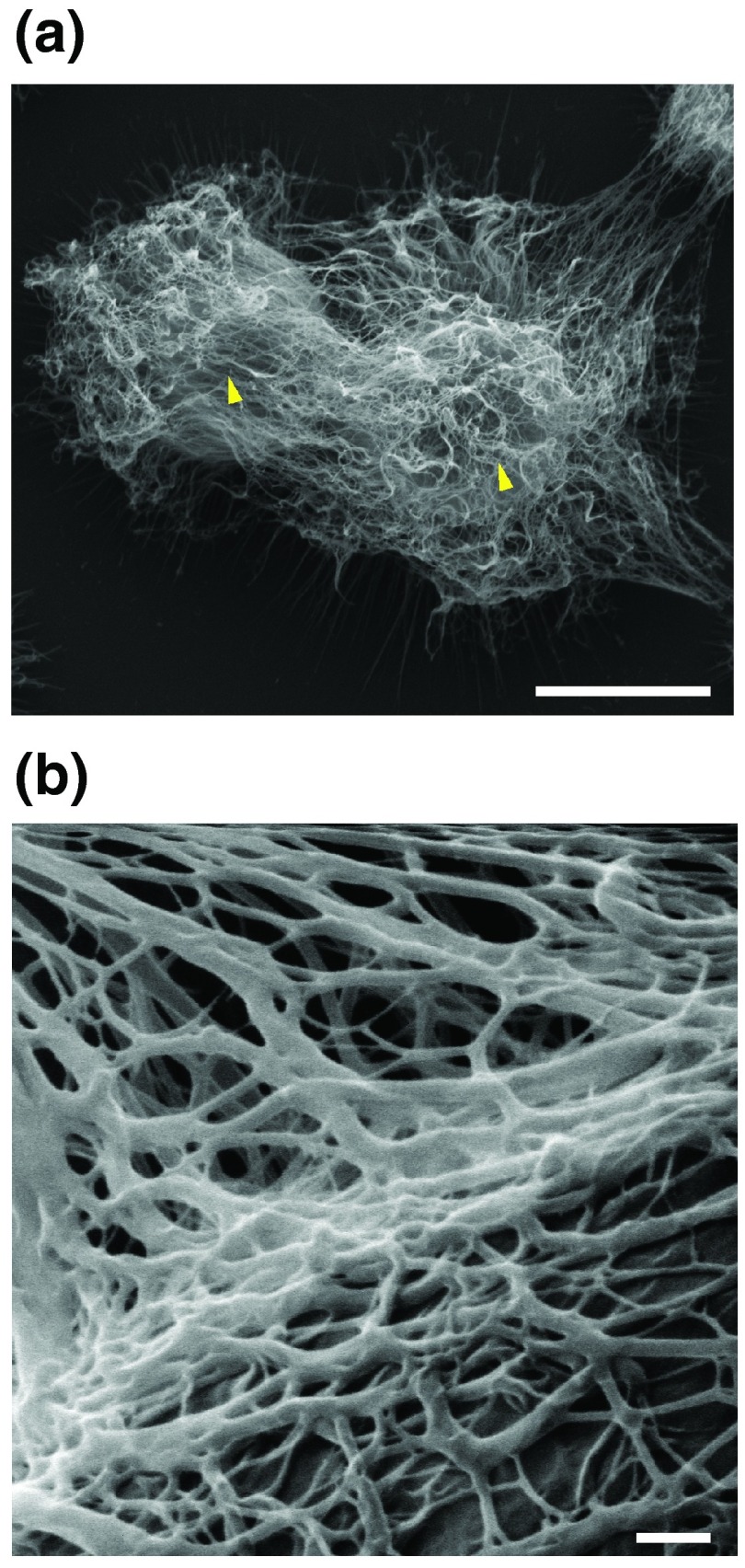
The network of polysaccharide fibers surrounding *Cryptococcus neoformans* cells. ( **a**) Conventional scanning electron microscopy showing the network of capsular fibers around two yeast cells marked by arrowheads. Bar, 5 μm. ( **b**) High-resolution scanning electron microscopy of the network of capsule fibers. Bar, 100 nm. The methods to generate the images are described in the study of Araújo *et al.* ^8^.

The ability of *C. neoformans* to cause disease is due in large part to its production of a capsule made up of fibers of two polysaccharides, glucuronoxylomannan (GXM) and glucuronoxylomannogalactan (GXMGal) ( Figure 1) ^8– 12^. The capsule is thought to protect cells from desiccation in the environment, and the GXM and GXMGal polysaccharides have immunomodulatory properties during disease in vertebrate hosts ^12, 13^. Acapsular mutants are generally avirulent, while many hypercapsular mutants show enhanced virulence in a mouse model of cryptococcosis ^12– 15^. Additional virulence traits include the formation of melanin in the cell wall, survival in macrophages and at host temperature, acquisition of limited nutrients in the host (e.g., iron), and production of extracellular enzymes such as urease and phospholipase B ^9, 16^.

The size of the capsule is influenced by a variety of host and environmental factors that include host tissue location, CO _2_ levels, serum, temperature, and the availability of nutrients such as iron and glucose ^10, 12^. Key signaling pathways that mediate the responses to some of these conditions include the cAMP/protein kinase A (PKA) pathway, the Hog1 stress-response pathway, the PKC pathway, and the pheromone response mitogen-activated protein kinase (MAPK) pathway ^12, 13^. As discussed below, a number of transcription factors (TFs) have been identified that respond to environmental conditions and that function downstream of the signaling pathways to regulate the capsule. In this review, we focus on recent studies that use high-throughput approaches and network analyses to identify new virulence traits and address the key question of how virulence is regulated. In particular, these studies are positioning known and newly identified TFs in regulatory networks that influence the ability of the fungus to produce the capsule and other virulence-related functions.

## Towards a whole-genome knockout collection for *C. neoformans*

Completion of the *C. neoformans* sequencing projects enabled a powerful approach to study regulatory gene networks: the systematic deletion and analysis of each predicted fungal gene ^17– 19^. This large-scale approach has very successfully been used before in the model yeast *Saccharomyces cerevisiae* ^20^. The *C. neoformans* genome encompasses 6967 predicted genes ^17^. Until 2008, only ~190 targeted gene deletion mutants had been constructed and analyzed (corresponding to 2.7% of the whole genome). In a landmark study for cryptococcal research, the laboratories of Hiten Madhani and Suzanne Noble began to systematically construct gene knockout mutants via biolistic transformation and homologous recombination ^18^. Target genes were not chosen randomly but rather selected based on two criteria: absence of the homologous gene in the non-pathogenic yeast *S. cerevisiae* and presence of predicted sequence motifs known to potentially contribute to fungal virulence. The number of generated mutants was impressive: 1180 distinct genes were successfully knocked out (representing 16.9% of the total genome). With this major mutant collection in hand, the Madhani and Noble groups then systematically analyzed each knockout mutant for its pathogenic potential during murine lung infection and for the three key cryptococcal virulence factors: production of melanin, elaboration of the polysaccharide capsule, and the capacity to grow at human body temperature ^18^. Approximately 30 mutants of the deletion collection had disruptions of predicted or known TFs. Of those, 15 mutants displayed aberrant virulence in mice and/or defects in virulence factor production. Specifically, the TFs Hcm1, Liv1, Liv2, Liv3, Liv4, Pdr802, Snt1, and Zap103 were exclusively required for normal lung infectivity while being dispensable for virulence factor production. These TFs are particularly interesting because identifying their mechanism of action may reveal additional, currently unknown virulence determinants in *C. neoformans*. The TFs Zap104 and Mbp102 were shown to be essential for both lung infection and melanin production. Additional TFs required for melanin formation included Mln1, Nhp6b01, Pan1, and Rim101. Finally, the TF Gat201 was demonstrated to be required for lung infectivity, capsule formation, and normal melanin synthesis (a *gat201Δ* mutant was shown to produce more melanin than the wild-type strain). The *gat201Δ* mutant produced no visible capsule via India ink staining and was completely avirulent in mice. By performing transcriptional profiling studies on a *GAT201*-overexpression strain, it was found that Gat201 regulates at least 567 genes, 25 of which encode TFs. This finding provided an initial clue to the emerging complexity of the regulatory network for virulence genes in *C. neoformans*.

Macrophages are key host immune cells that form one of the first lines of defense against invading pathogens ^21^. Most pathogenic microbes are efficiently taken up and killed by these immune cells. Some pathogens, like *C. neoformans*, however, have evolved mechanisms of resisting uptake and/or killing by macrophages, and the capsule has been described as the main antiphagocytic factor in this process ^22^. An unexpected finding for the role of Gat201 emerged from studies on the interaction of a *gat201Δ* mutant with murine macrophages. That is, Gat201 regulates a previously unknown antiphagocytic mechanism that is independent of capsule production ^18, 23^. Transcriptional profiling studies on a *gat201Δ* mutant under macrophage culture conditions established that Gat201 regulates approximately 1100 genes ^23^. Promoter binding studies subsequently revealed that 126 genes were directly bound by Gat201. Among these genes were seven TFs, again shedding light on an intricate transcriptional regulation network for virulence in *C. neoformans*. Interestingly, these TFs included the major regulator of iron uptake/utilization and virulence, Cir1, and another GATA family TF, Gat204 ^23, 24^. Cir1 was previously characterized as a key link between iron sensing and capsule formation ^24^. Gat204 was found to be largely responsible for the capsule-independent antiphagocytic effect originally observed in the *gat201Δ* mutant, establishing a functional and mechanistic link between those two novel TFs. It will be interesting to analyze the targets of Gat204 in the future.

## Analysis of a targeted collection of transcription factor mutants

Yong-Sun Bahn and his colleagues recently constructed a high-quality collection of 322 signature-tagged mutants for 155 putative TFs in an effort to understand how these factors influence specific phenotypic traits in *C. neoformans* ^25^. The TFs were selected from a total of 178 predicted DNA-binding domain (DBD) protein candidates from the *C. neoformans* serotype A (strain H99) genome database (version 7). The collection of deletion mutants was functionally analyzed for distinct *in vitro* and *in vivo* phenotypes related to virulence, and this approach generated an impressively large phenome data set for 32 growth conditions ( http://tf.cryptococcus.org) ^25^. Importantly, phenotypes were uncovered for 93% of the TFs, and many TFs were characterized for the first time and linked to phenotypic traits relevant to virulence. In particular, TFs that influenced the major virulence factors were identified including nearly 32% (49) that were linked to the regulation of capsule production. These included previously identified capsule regulators such as Ada2, Gat201, Atf1, and Mbs1, as well as newly characterized TFs whose deletion significantly decreased (Zap104, Yap1, and Rds2) or increased (Hob7, Clr3, and Fzc51) capsule size.

TFs that influenced other virulence traits included ~17% (27) that were related to melanin production, such as Fzc8, Hob1, and Bzp4 that had reduced melanin upon deletion and Hlh1, Hlh2, Yap1, and Fzc1 that had increased melanin production ^25^. Although some of the identified TFs were found to positively or negatively regulate the expression of *LAC1*, encoding the laccase enzyme responsible for melanin synthesis in *C. neoformans*, some of the TFs were not linked to this activity. These TFs join previously characterized regulators of melanization including Cuf1, Ste12, Mbs1, Skn7, and Atf1 ^25^. TFs were also identified that influenced growth and thermotolerance. For example, deletion mutants of 17% (27) of the TFs showed growth defects only at 37–39°C (e.g., *hxl1Δ*, *crz1Δ*, *atf1Δ*, *ada2Δ*, *hap1Δ*, *aro80Δ*, *usv101Δ*, *fzc31Δ*, *fzc30Δ*, *fzc1Δ*, *miz1Δ*, *apn2Δ*, *gat6Δ*, *mbs2Δ*, *sre1Δ*, and *ert1Δ*), and deletion of some TFs showed increased thermotolerance at 39°C (e.g., *mln1Δ*, *mcm1Δ*, and *fzc46Δ*). Extracellular enzymes also contribute to *C. neoformans* virulence, and urease activity, for example, contributes to fungal invasion of the central nervous system. In the analysis of Jung *et al*. ^25^, 19 out of 155 (12%) TFs were related to the positive or negative regulation of urease production. These included two (Hlh1 and Zap104) whose loss significantly reduced urease production and six with the opposite influence (Atf1, Rim101, Fzc1, Skn7, Fzc14, and Hob7).

The TF deletion collection was also employed to characterize antifungal drug susceptibility and virulence in animal models ^25^. For example, resistance to the drugs amphotericin B, fluconazole, and flucytosine that are commonly used to treat cryptococcosis was linked to 5% (8), 13% (20), and 17% (27) of the identified TFs, respectively. Two of the identified TFs, Hob1 and Sre1, regulate sterol biosynthesis that is relevant to susceptibility to fluconazole and amphotericin B. Additionally, the *hob1Δ* and *sre1Δ* mutants were significantly impacted in their response to most of the environmental stress conditions analyzed, thus suggesting more general roles. Overall, the analysis of the TF deletion collection will support efforts to understand the mechanisms that lead to the emergence of resistant strains of *C. neoformans*. The value of TFs as novel targets for antifungal drugs has recently been reviewed ^26^. The large-scale virulence assays performed with 155 TF mutants used the insect model system *Galleria mellonella* and a signature-tagged mutagenesis approach in a mouse model. More than 30 novel TFs implicated in virulence were identified in both assays. Interestingly, some of these TF deletion mutants did not show conspicuous phenotypic traits related to their virulence, suggesting potential regulation of unidentified virulence factors.

In general, the relationship of the structurally diverse TFs and their influence on the numerous phenotypic traits, as determined by Jung *et al.* ^25^, generate a wealth of new hypotheses and opportunities to gain insights into the regulation of *C. neoformans* virulence. The groups of Yong-Sun Bahn and Insuk Lee have also taken a computational approach to better understand the complexity of the biological pathways of *C. neoformans* by creating a genome-scale co-functional network called CryptoNet ( www.inetbio.org/cryptonet) ^27^. This integrative systematic analysis tool is based on 14 distinct types of large-scale data covering co-functional links of more than 5000 genes from the *C. neoformans* genome. Using gene prioritization algorithms such as *guilt-by-association* and *context-associated hub*, it was possible to identify novel genes involved in virulence and drug resistance. Overall, this type of network analysis combined with functional genomic data and deletion mutant collections is a powerful tool to discover the regulatory factors and target genes involved in *C. neoformans* virulence.

## A network approach to model capsule regulation

To reveal genes whose expression is associated with capsule elaboration, the laboratories of Tamara Doering and Michael Brent analyzed transcript abundance under eight different capsule-inducing and non-inducing conditions and identified 880 genes whose transcription significantly correlated with capsule size ^28^. Most positively correlated genes were associated with responses to stress, whereas most negatively correlated genes were involved in mitochondrial function and ribosome biogenesis. These results are consistent with a recent proteomic study in which proteins associated with ribosome biogenesis and translation were less abundant upon induced expression of the catalytic subunit of PKA (Pka1) that resulted in a large capsule compared to a Pka1-repressed condition ^29^. Haynes *et al.* ^28^ selected an *ADA2* homologue, encoding a putative protein in the SAGA histone acetylation complex, for further analyses based on a strong correlation between expression and capsule size. RNA-seq combined with ChIP-seq allowed identification of targets of Ada2. The effect of Ada2 on capsule production appeared to be indirect by affecting the histone acetylation near the transcription start sites of a variety of genes including several previously identified to influence capsule: *HXT1*, *CPL1*, *UGT1*, *GAT201*, and *STE2*.

In subsequent work, Maier *et al*. ^30^ developed and demonstrated a feasible, efficient, and novel workflow to identify transcription factors involved in capsule production. They first applied a bioinformatics tool, NetProphet ^31^, to gene expression profiles of strains with deletions of genes encoding regulators. This tool, which combines data from both co-expression and differential expression, ranks all possible TF-target interactions. They then used a second algorithm, PhenoProphet, to predict new TFs associated with capsule phenotypes (based on whether the target genes of each TF were enriched for those that influence capsule). Deletion of the genes encoding the predicted TFs and subsequent transcription profiling generated data to be fed through NetProphet, resulting in refined network models and additional predicted TFs associated with the phenotype of interest (i.e., capsule size). Experimental validation showed that all of the TFs most confidently predicted to influence capsule phenotype did regulate capsule thickness (either absolute value or variability), and many also regulated fungal virulence.

**Figure 2.**
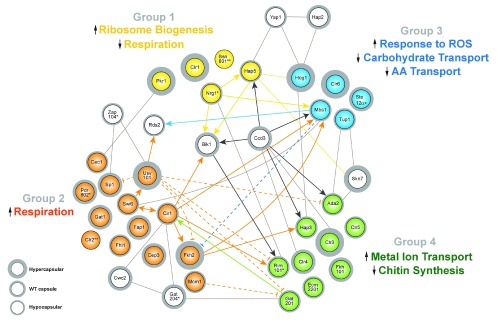
A network of transcription factors affecting capsule size in *Cryptococcus neoformans*. The connections between transcription factors and the information on capsule sizes for the corresponding mutants were adapted from Maier *et al.* ^30^, Gish *et al.* ^32^, Jung *et al.* ^25^, and CryptoNet ( www.inetbio.org/cryptonet) ^27^. The colors of the transcription factors indicate the different groups, and the white factors do not clearly fit in any of the four groups. Small up and down arrows indicate upregulation and downregulation of functions in the different groups, respectively. *Capsule size may differ between specific studies owing to differences in growth conditions. **These mutants have hypervariable capsule sizes. Abbreviations: AA, amino acid; ROS, reactive oxygen species, WT, wild-type.

Maier *et al.* ^30^ also examined the transcriptional dynamics of capsule induction by RNA-seq on wild-type cells upon transfer from rich media to capsule-inducing conditions and discovered that genes involved in protein translation were down-regulated, whereas genes with functions in specific amino acid synthesis and protein degradation were induced, consistent with other studies ^28, 29^. Moreover, based on temporal expression patterns of TFs during capsule induction, Maier *et al*. ^30^ were able to build a hierarchical network of four groups of TFs and regulatory proteins such as signaling components. Group 1 TFs sit on top of the hierarchy, with expression levels slightly increased within 90 minutes of transfer and sharply decreased over the next 24 hours. These TFs include activators of ribosome biogenesis, repressors of mitochondria-encoded respiration genes, and repressors of a cluster of capsule-involved genes. Group 2 TFs are repressed by TFs of Group 1, hence having expression profiles opposite to the factors in Group 1. These TFs regulate mitochondria-encoded respiration genes and genes involved in capsule formation that do not encode TFs. Group 3 regulators have similar expression profiles to Group 2 with decreased expression within the first 90 minutes followed by increased transcript levels, except that the expression levels never recovered to initial abundance. Group 3 regulators appear to activate genes in response to reactive oxygen species and to repress genes encoding carbohydrate and amino acid transporters. Group 4 regulators do not regulate the genes in other groups and therefore sit at the bottom of the hierarchy. The expression of these genes steadily increased within 24 hours upon transferring from rich media to capsule-inducing conditions. Figure 2 presents a schematic of the network defined by the four groups identified by Maier *et al*. ^30^, with representative genes within each group and additional TFs and regulatory proteins from other studies ^18, 25, 27, 32^. It is noteworthy that each study employed specific capsule-inducing approaches and the structure of the network may therefore be condition dependent. Nevertheless, the approach in combination with PhenoProphet and temporal expression pattern analysis provides a powerful tool for building regulatory networks.

In a follow up study, Gish *et al*. ^32^ demonstrated the requirement of transcription and translation for capsule induction using the inhibitors 1,10-phenanthroline and cycloheximide, respectively, and argued that the dominant regulatory control occurs at the transcriptional level. The influence of cycloheximide and proteostasis on capsule production was also recently demonstrated in a proteomics study ^29^. Gish *et al*. ^32^ went on to focus on building regulatory networks centered on the TF Usv101. They found that the *usv101*Δ mutant showed attenuated virulence in an intranasal mouse model of cryptococcosis, although it also had a hypercapsular phenotype. Based on TF profiling data presented in Maier *et al.* ^30^, the hierarchy position of Usv101 in the capsule regulatory cascade was determined to be high, with only Swi6 located above it. The function of Usv101 appears to converge with the cAMP/PKA signaling pathway to regulate the *GAT201-*encoded TF mentioned above ^23^. The direct targets of Usv101 were determined by identification of its binding motif through temporal expression pattern analysis via RNA-seq in combination with ChIP-seq ^30^. Subsequent examination of mutants in the target genes identified those with abnormal capsule. A total of 19 Usv101 targets involved in capsule formation were identified, and 13 of these were repressed by Usv101. These included six TF genes: *FKH2*, *CLR2*, *CLR1*, *GAT201*, *RIM101*, and *SP1*. Deletion mutants for the latter three showed dramatically reduced capsule thickness, consistent with the repressor role of Usv101. The relationship between Usv101 and *GAT201*, *RIM101*, as well as *SP1* was further confirmed with double knockout strains. Interestingly, Usv101 represses the expression of only one non-TF target gene, *UXS1*, that is involved in capsule formation. *UXS1* encodes a UDP-xylose synthase that decarboxylates UDP-glucuronic acid to form UDP-xylose, both of which contribute to glycan synthesis and GXM. Usv101 also regulates melanin production, resistance to salt, and a number of genes involved in the synthesis of cell wall components.

## Conclusion and future perspectives

Recent studies provide a simple answer to the question of how capsule and other virulence factors are regulated in *C. neoformans*: it's complicated! That is, the emerging view is one of a remarkably complex network of TFs and other regulatory factors that are interconnected with metabolic and mitochondrial functions, ribosome biogenesis, and the response to stress. This view provides an excellent framework to guide future work, and many tasks and questions remain. One key task is to complete the whole-genome knockout collection, and the Madhani laboratory is currently working on this goal. Approximately 2200 additional genes have been deleted and the mutant strains were made publically available to the research community through the Fungal Genetics Stock Center ( http://www.fgsc.net/). The deletion collection will facilitate further studies of functions that influence capsule and virulence as well as chemical genetic studies to identify drug targets, as already initiated by the Madhani group ^33^. It will be important to extend the collection to include essential genes and to develop mutational strategies for their analysis ^34, 35^. It will also be important to dig deeper into the regulatory hierarchy to identify targets and sort out the more complicated regulatory connections to understand virulence phenotypes. For example, more analysis is needed to explain why a mutant lacking the TF Cdc3 does not have a capsule phenotype but the protein activates the expression of four TFs that do influence the capsule ^30^. Finally, it will be interesting to examine whether the emerging regulatory network defined *in vitro* also functions similarly during growth in mammalian hosts. Answers to these questions will further refine the emerging model of regulation of virulence and hopefully provide insights to support therapeutic intervention.

## Abbreviations

GXM, glucuronoxylomannan; GXMGal, glucuronoxylomannogalactan; PKA, protein kinase A; TF, transcription factor; DBD, DNA binding domain.
